# Free Fasciocutaneous versus Muscle Flaps in Lower Extremity Reconstruction: Implications for Functionality and Quality of Life

**DOI:** 10.1055/a-2483-5388

**Published:** 2024-12-20

**Authors:** Emile B. List, Brett A. Hahn, Shan S. Qiu, Tim de Jong, Hinne A. Rakhorst, Elfi M. Verheul, Wiesje Maarse, J. Henk Coert, David D. Krijgh

**Affiliations:** 1 Department of Plastic, Reconstructive and Hand Surgery, University Medical Center Utrecht, Utrecht, The Netherlands; 2 Department of Plastic, Reconstructive and Hand Surgery, Maastricht University Medical Center, Maastricht, The Netherlands; 3 Department of Plastic, Reconstructive and Hand Surgery, Radboud University Medical Center, Nijmegen, The Netherlands; 4 Department of Plastic, Reconstructive and Hand Surgery, Medisch Spectrum Twente, Enschede, The Netherlands

**Keywords:** lower leg, free flap, reconstruction

## Abstract

**Background:**

Free tissue transplantations are commonly used to treat complex lower extremity defects caused by trauma, vascular disease, or malignancy, particularly when vital structures are exposed. This study aimed to expand the knowledge on patient-reported outcomes by comparing fasciocutaneous and muscle flaps, with the goal of facilitating patient counseling. Additionally, patient-level risk factors associated with decreased functioning and health-related quality of life were identified.

**Methods:**

This retrospective, cross-sectional, multicenter study included patients who underwent microsurgical lower extremity reconstruction using free fasciocutaneous or muscle flaps between 2003 and 2021, with a minimum follow-up of 12 months. Data were collected from medical records, pain scores, Short-Form 36 (SF-36), and Lower Extremity Functional Scale (LEFS). Mean scores were compared between flap types and predictors of LEFS, SF-36 mental component summary (MCS), and SF-36 physical component summary (PCS) scores were determined using a backward stepwise regression model.

**Results:**

Of the 206 patients eligible, 100 (49%) were included in the retrospective part. A total of 89 (43%) responded to the questionnaires, with 62 treated using a fasciocutaneous flap and 27 with a muscle flap. No significant differences in total LEFS, SF-36 PCS, or MCS scores were found between the two flap type. Pain was a significant predictor of decreased functional outcomes for both flap types and was also linked to poorer mental health in patients treated with fasciocutaneous flaps. Other predictors of low patient-reported outcome scores included obesity, diabetes, poorer mental health, and a follow-up of less than 2 years.

**Conclusion:**

Patients treated with fasciocutaneous and muscle flaps experience comparable levels of functionality and quality of life after surgery. Flap selection should be based on defect characteristics, along with the surgeon's individual skills and preferences. A comprehensive approach that considers physical comorbidities, pain, and mental health is essential, as these factors significantly impact patient functionality and quality of life.

Over the last decades, free flap reconstructions have become daily practice for many microsurgeons. Complex lower extremity defects exposing vital structures due to trauma, vascular disease, or malignancy are important parts of practices of plastic surgeons. When primary closure, skin grafts, or local flaps reconstructions are no option to close the defect, a free tissue transplantation is necessary to salvage the limb, avoid amputation, and restore the leg to its most functional state. Main parts of the armamentarium of the reconstructive surgeons are either fasciocutaneous or muscle flaps. In terms of limb salvage and flap loss both types of flaps—fasciocutaneous or muscle—have been proven to be safe and have comparable outcomes. 1 2 3

Discussion on flap selection remain part of many conferences and manuscripts. Practice variation is accepted and is often based on surgeon, practice country, or groups of collaborative surgeons. Previous studies have conducted comparative analyses of free fasciocutaneous and muscle flaps in terms of, for example, limb salvage, although a consensus which type of flap to choose is lacking. However, appreciating patient-related perspectives and risk factors contributing to poorer outcomes may help and improve decision-making.

Therefore, the aim of this study was to gain insight into patient-reported outcomes of lower extremity free flap reconstructions and to compare the outcomes of fasciocutaneous to muscle flaps, which will help in counseling patients. Additionally, the study aimed to identify patient-level risk factors associated with decreased functionality and health-related quality of life (HRQOL).

## Methods

### Study Design

A retrospective and cross-sectional multicenter study was done using data collected from patients who underwent a microsurgical lower extremity reconstruction using a fasciocutaneous or muscle free flap. Patient records from the UMC Utrecht, Maastricht UMC, Medisch Spectrum Twente, and Radboud UMC of patients operated between January 2003 and December 2021 were used. Ethical approval for this study was obtained from the Medical Research Ethics Committee (reference number 22-761, METC NedMec).

### Patient Selection

Patients were eligible for inclusion if they met the following criteria: (1) they underwent a microsurgical lower extremity reconstruction using a free fasciocutaneous or free muscle flap, (2) had an age of 16 years or older at the time of surgery, and (3) had a follow-up of at least 12 months after the reconstruction. Exclusion criteria were a mental or physical inability to read, understand, and/or complete the questionnaires. All patients were recruited between October and December 2022. Eligible patients were informed about the study by phone. Written informed consent was obtained from all participants before the study. Patients completed the questionnaires using Castor EDC, a secured web-based clinical data management platform. 4

### Demographics

Patient-related characteristics and surgery results were recorded from the electronic medical records. These included gender, age, body mass index (BMI), comorbidities, and medical history. Surgery-related characteristics included time since reconstruction, cause and location of defect, free flap used, and Gustilo classification. Surgery results included flap revision and flap/donor site complications. Complications that were recorded from the medical records were flap/donor site necrosis, vascular insufficiency, wound infection, wound dehiscence, fistula formation, seroma, hypertrophic scar, and postoperative hemorrhage.

### Patient-Reported Outcomes Measures

Patient-reported outcomes were measured using two questionnaires in Dutch and a pain-score. All patient-reported outcomes measures were validated and translated in Dutch. General health was determined using the 36-Item Short-Form Health Survey (SF-36), a widely used HRQOL questionnaire consisting of 36 items divided into eight scales. 5 The scores range from 0 to 100, with higher scores representing better physical and mental well-being. The summary scores physical component summary (PCS) and mental component summary (MCS) were calculated from the eight domains using normative data from a Dutch population combined with U.S. factor score coefficients. 6 7 Lower extremity function was assessed using the Lower Extremity Functional Scale (LEFS), a questionnaire including 20 items on a 5-point scale (0–4). 8 9 Total LEFS score ranges from 0 to 80, with higher scores indicating greater level of functional status. Pain was evaluated using a 5-point Likert scale, with scores ranging from 0 (No pain or discomfort) to 4 (Extreme pain or discomfort).

### Patient-Level Risk Factors

The binary variables that were examined to determine their correlation with poorer LEFS, SF-36 MCS, and SF-36 PCS scores included gender, smoking status, hypertension, cardiovascular disease, diabetes mellitus, obesity, primary or secondary reconstruction, surgical complication, level of defect, oncologic defect, traumatic defect, recent or past trauma, and pain. The variable mental health was included in the LEFS and SF-36 models only; the groups were formed based on SF-36 MCS scores with reference to the lower quartile. The variable smoking status was divided into two groups: “active smokers” and “nonsmokers.” The variable surgical complication was divided into two groups: patients who endured any type complication and those who did not. Level of defect was divided in the groups “knee to lower leg” and “ankle to foot.” Pain was defined as “yes” if it was rated as moderate or worse and “no” if it was rated as no pain or slight pain. The group “recent trauma” encompassed patients who sustained a traumatic event as cause of the defect that occurred within a 6-week period prior to the reconstruction, whereas “past trauma” consisted of patients whose traumatic event had happened earlier.

### Statistical Analysis

For analyses, the patients were divided into two groups based on the free flap used for reconstruction: fasciocutaneous and muscle flap. Patient and surgery-related variables were summarized using descriptive statistics and presented as means or medians with interquartile ranges or numbers with percentages. Mean LEFS scores and SF-36 health-related quality-of-life domains were compared between patient groups according to free flap type using one-way analysis of variance. Stepwise regression of multiple demographic and postoperative complication variables were conducted using backward selection. All variables with a value of *p* < 0.10 were included in the predictive model. Predictors of LEFS, SF-36 MCS, and SF-36 PCS scores according to free flap type with a value of *p* < 0.05 were considered statistically significant. Statistical analyses were conducted using R Statistical Software (v4.3.1; R Core Team 2023).

## Results

### Patient and Surgery Characteristics

A total of 206 patients were identified who had received a microsurgical leg reconstruction using a fasciocutaneous or muscle free flap. A total of 100 (49%) patients were included in the retrospective part of the study and 89 (43%) responded to the questionnaires. All patient and surgery characteristics are shown in Tables 1 and 2 . Unfortunately, of the 206 patients, 79 patients were unreachable, 27 denied participation or did not return the consent form.

**Table 1:** Patient characteristics per group based on free flap used

	Fasciocutaneous flap *N* = 69	Muscle flap *N* = 31	*p* -Value
Sex, *N* (%)			
Male	50 (73)	24 (77)	0.606
Female	19 (28)	7 (23)	
Age in years, median (IQR)	59 (48–66)	61 (43–70)	0.777
BMI, median (IQR)	26.6 (23.6–29.2)	27.8 (24.4–29.4)	0.633
Comorbidities, *N* (%)			
Current smoker	8 (12)	7 (23)	0.158
Smoking history	25 (36)	6 (19)	0.093
Hypertension	17 (25)	7 (23)	0.826
Cardiovascular disease	9 (13)	4 (13)	0.985
Diabetes mellitus	6 (9)	1 (3)	0.326
Hospital, *N* (%)			
Maastricht UMC	39 (57)	1 (3)	<0.001
Medisch Spectrum Twente	4 (6)	3 (10)	
Radboud UMC Rotterdam	11 (16)	6 (20)	
UMC Utrecht	15 (22)	21 (68)	

**Table 2:** Surgery characteristics per group based on free flap used

	Fasciocutaneous flap *N* = 69	Muscle flap *N* = 31	*p* -Value
Years since surgery, median (IQR)	4.1 (2.5–5.9)	6.0 (2.8–7.9)	0.464
Location defect, *N* (%)			
Knee	2 (3)	2 (6)	0.063
Lower leg	41 (59)	21 (68)	
Ankle	6 (9)	6 (19)	
Heel	8 (12)	2 (6)	
Foot	12 (17)	0 (0)	
Cause of defect, *N* (%)			
Recent trauma (within 6 wk)	19 (28)	6 (19)	0.620
Trauma in the past	31 (45)	21 (68)	
Vascular disease	3 (4)	1 (3)	
Malignancy	10 (15)	1 (3)	
Other cause	6 (9)	2 (7)	
Type of free flap, *N* (%)			
Gracilis flap	0 (0)	15 (48)	
Latissimus dorsi flap (LD)	0 (0)	12 (39)	
Rectus abdominis flap	0 (0)	3 (10)	
Vastus lateralis flap	0 (0)	1 (3)	
Anterolateral thigh flap (ALT)	48 (70)	0 (0)	
Free radial forearm flap (FRFF)	14 (20)	0 (0)	
Deep inferior epigastric perforator flap (DIEP)	2 (3)	0 (0)	
Parascapular flap (PS)	2 (3)	0 (0)	
Superficial circumflex iliac artery perforator flap (SCIP)	2 (3)	0 (0)	
Thoracodorsal artery perforator flap (TDAP)	1 (1)	0 (0)	
Gustilo classification, *N* (%)			
2	5 (7)	1 (3)	0.349
3A	5 (7)	0 (0)	
3B	4 (6)	5 (16)	
3C	3 (4)	0 (0)	
3 (unspecified)	6 (9)	1 (3)	
Unknown/does not apply	46 (67)	24 (77)	

### Complications

Partial flap necrosis was observed in five patients (7%) who received a fasciocutaneous flap and in four patients (13%) in the muscle group. Five patients (7%) in the fasciocutaneous group and three (10%) in the muscle group required revision surgery. Five individuals (7%) had complications at the donor site after undergoing a fasciocutaneous flap reconstruction, four patients (13%) after receiving a muscle flap. All complications are shown in Table 3 .

**Table 3:** Surgery complications per group based on free flap used

	Fasciocutaneous flap *N* = 69	Muscle flap *N* = 31	*p* -Value
Flap revision, *N* (%)			
Yes	5 (7)	3 (10)	0.682
Flap complications, *N* (%)			
Any complication	15 (22)	6 (19)	0.789
Partial flap necrosis	5 (7)	4 (13)	
Vascular insufficiency	4 (6)	3 (10)	
Wound infection	2 (3)	1 (3)	
Wound dehiscence	7 (10)	0 (0)	
Fistula formation	0 (0)	1 (3)	
Complication donor site, *N* (%)			
Any complication	5 (7)	4 (13)	0.366
Partial donor site necrosis	1 (1)	0 (0)	
Wound infection	0 (0)	1 (3)	
Wound dehiscence	3 (4)	0 (0)	
Seroma	0 (0)	2 (6)	
Hypertrophic scar	1 (1)	0 (0)	
Postoperative hemorrhage	0 (0)	1 (3)	

### Patient-Reported Outcomes

The questionnaires were completed by 89 patients, of which 62 were treated with a free fasciocutaneous flap and 27 with a muscle flap. The median time since the reconstruction was 4.3 years (range: 1.0–19.1). Table 4 presents the SF-36 scores per group; no significant differences were seen between fasciocutaneous and muscle flaps regarding the eight scales and two component scores. Table 5 shows the scores for all LEFS questions per group; no significant differences were seen between the groups.

**Table 4:** Mean 36-Item Short-Form Health Survey scores per group based on free flap used

Variables	Fasciocutaneous flap *N* = 62	Muscle flap *N* = 27	*p* -Value
Physical functioning	67.3 ± 24.3	65.0	0.671
Role limitation due to physical health	57.3 ± 43.5	65.7 ± 39.3	0.387
Role limitation due to emotional problems	72.0 ± 41.9	79.0 ± 37.2	0.458
Vitality	66.0 ± 18.7	64.1 ± 14.3	0.641
Mental health	77.7 ± 14.0	75.3 ± 15.7	0.460
Social functioning	77.0 ± 22.4	75.0 ± 19.0	0.685
Bodily pain	65.8 ± 24.8	69.9 ± 19.4	0.447
General health perception	63.5 ± 20.7	63.3 ± 17.7	0.977
Physical component summary	43.6 ± 10.2	44.4 ± 9.6	0.726
Mental component summary	50.4 ± 9.8	49.9 ± 9.5	0.835

Note: All values are presented as means ± standard deviation.

**Table 5:** Mean Lower Extremity Functional Scale scores per group based on free flap used

Activities	Fasciocutaneous flap *N* = 62	Muscle flap *N* = 27	*p* -Value
A. Any of your usual work, housework, or school activities	2.8 ± 1.2	3.1 ± 0.8	0.155
B. Your usual hobbies, recreational, or sporting activities	2.6 ± 1.3	2.7 ± 1.1	0.654
C. Getting into or out of the bath	3.2 ± 1.1	3.2 ± 1.1	0.959
D. Walking between rooms	3.4 ± 0.9	3.4 ± 0.6	0.932
E. Putting on your shoes or socks	3.4 ± 0.9	3.1 ± 1.0	0.176
F. Squatting	2.3 ± 1.5	2.1 ± 1.4	0.625
G. Lifting an object, like a bag of groceries from the floor	3.4 ± 0.9	3.3 ± 0.9	0.774
H. Performing light activities around your home	3.4 ± 0.8	3.4 ± . 0.6	0.801
I. Performing heavy activities around your home	2.3 ± 1.1	2.2 ± 1.2	0.893
J. Getting into or out of a car	3.3 ± 0.8	3.4 ± 0.8	0.719
K. Walking two blocks	3.3 + 0.9	3.3 ± 0.9	0.996
L. Walking 1 mile	2.5 ± 1.3	2.6 ± 1.3	0.722
M. Going up or down 10 stairs (approximately one flight of stairs)	3.0 ± 1.0	3.1 ± 0.9	0.528
N. Standing for 1 hour	2.3 ± 1.3	2.3 ± 1.3	0.797
O. Sitting for 1 hour	3.6 ± 0.8	3.6 ± 0.8	0.892
P. Running on even ground	1.0 ± 1.3	0.4 ± 0.9	0.065
Q. Running on uneven ground	0.7 ± 1.2	0.3 ± 0.6	0.099
R. Making sharp turns while running fast	0.8 ± 1.3	0.4 ± 0.9	0.098
S. Hopping	1.0 ± 1.3	1.0 ± 1.3	0.956
T. Rolling over in bed	3.6 ± 0.8	3.5 ± 0.8	0.461
LEFS total score (out of 80)	51.7 ± 15.6	50.4 ± 12.4	0.703

Notes: All values are presented as means ± SD. Scores ranging from 0 to 4 with 0 indicating extreme difficulty or inability and 4 indicating no difficulty to perform activity.

### Patient-Level Risk Factors

In the multivariable regression model for the fasciocutaneous flaps, the variables BMI, pain, diabetes, mental health, and years since surgery were found to be significant predictors of several patient-reported outcome scores ( Table 6 ). Pain reported as “moderate or worse” was a significant predictor of lower MCS, PCS, and total LEFS scores. A BMI above or equal to 30 was a significant predictor of low MCS scores. The presence of diabetes was found to be a significant predictor of decreased PCS scores. An MCS score in the lower quartile, and a follow-up of 2 years or less after surgery were significant predictors of lower total LEFS score. The multivariable regression model for muscle flaps showed that only the variable pain was a significant predictor of decreased total LEFS scores ( Table 6 ).

**Table 6:** Overview of significant variables by multivariable analyses for fasciocutaneous and muscle flaps

Variables	Patient responses	Mean score	Beta	95% Confidence intervals		
				Lower	Upper	*p* -Value
Fasciocutaneous flap						
MCS						
BMI	≥ 30 ( *n* = 12)	45.0	−6.305	−12.120	−0.489	0.034
	< 30 ( *n* = 50)	51.7				
Pain a	Yes ( *n* = 25)	46.8	−5.046	−9.763	−0.329	0.036
	No ( *n* = 37)	52.9				
PCS						
Diabetes	Yes ( *n* = 5)	37.3	−10.024	−18.301	−1.748	0.018
	No ( *n* = 57)	44.1				
Pain a	Yes ( *n* = 25)	38.0	−10.316	−14.886	−5.745	<0.001
	No ( *n* = 37)	47.4				
LEFS						
MCS score b	Low ( *n* = 16)	44.0	−8.073	−15.775	−0.370	0.040
	High ( *n* = 46)	53.9				
Pain a	Yes ( *n* = 25)	43.1	−11.741	-18.816	-4.612	0.001
	No ( *n* = 37)	57.5				
Years since surgery	< 2 y ( *n* = 8)	40.5	−10.891	−21.146	−0.637	0.038
	> 2 y ( *n* = 54)	53.2				
Muscle flap						
LEFS						
Pain a	Yes ( *n* = 13)	43.8	−12.725	−21.303	−4.148	0.005
	No ( *n* = 14)	56.6				

Abbreviations: BMI, body mass index; LEFS, Lower Extremity Functional Scale; MCS, mental component summary; PCS, physical component summary.

a Pain was defined as “yes” if rated as moderate or worse and “no” if rated as no pain or slight pain.

b MCS score, low (≤) and high (>) defined with reference to the lower quartile.

The variables gender, smoking status, hypertension, cardiovascular disease, primary or secondary surgery, surgical complication, oncologic defect, traumatic defect, and recent or past trauma were not significant predictors of decreased MCS, PCS, and LEFS scores.

## Discussion

Microsurgical lower extremity free flap reconstructions for complex defects at the extremities have become daily practices for many microsurgeons. Free flap surgery is developing into a highly reliable option for the reconstructive surgeons. Flap selection follows the principle to strive for optimal functional outcomes and decrease donor site morbidity. The selection of flap type is a subject of continued debate and fills programs of conferences and tables of contents of many journals. This cross-sectional study compared free flap reconstructions using fasciocutaneous with reconstructions using muscle flaps. Patient-reported functionality nor HRQOL results were able to show significant differences between both options. Risk factors that contribute to poorer results were determined, outcomes indicated that pain was significantly related to decreased functional outcomes for both fasciocutaneous and muscle flaps and to lower mental and physical scores of patients treated with fasciocutaneous flaps. In addition, regarding fasciocutaneous flaps a BMI above 30, diabetes, poorer mental health, and a follow-up of less than 2 years were predictors of lower scores.

Prior studies comparing fasciocutaneous and muscle flaps for functionality have found comparable results. Cho et al reported no difference between fasciocutaneous and muscles flaps regarding return to ambulation (defined as time to full weight-bearing status) in patients treated for acute and chronic traumatic wounds. 10 In the treatment of distal third and ankle traumatic open tibial fractures, Yazar et al also found comparable functional outcomes if selected for the appropriate defects. 11 They stated that free muscle flaps are more suitable for major tridimensional defects, whereas both flaps are equally effective in smaller fractures. Black et al conducted a comparison between free fasciocutaneous anterolateral thigh flaps versus vastus lateralis muscle flaps for the management of chronic wounds of the lower extremities and observed similar ambulation rates. 12 Our findings on functionality are in line with these studies but have now been examined and confirmed through the patient's perspective. The selection of free flap type should be based on defect characteristics in combination with the individual skills and preferences of the plastic surgeon.

Pain continues to be an important factor for patients and has shown to be a prognostic indicator for poorer outcomes in both cohorts within the scope of this study. Egeler et al found similar results in a cohort of patients treated with lower extremity free flap reconstructions for solely traumatic injuries. 13 They concluded that after a mean follow-up of 10 years, chronic pain was an independent predictor for lower scores of both PCS and LEFS. Harries et al reported data on pain after lower extremity injuries requiring flap coverage and the impact on patient's quality of life. 14 They stated that persistent pain following the reconstruction significantly affects the patient's quality of life, specifically their enjoyment of life and the capacity to work and walk. A total of 43% of the patients in our cohort reported that they experienced pain of at least moderate severity. Effectively addressing these symptoms has the potential to enhance the functionality of these individuals. An interdisciplinary approach in the treatment of chronic pain has demonstrated a significantly improvement in physical function. 15

Obesity is characterized as having a BMI that exceeds 30. This condition was in our cohort a predictor of decreased mental health for patients treated with a fasciocutaneous free flap. This relationship is frequently explored in the literature, concluding that obesity has a major impact on the mental well-being of individuals. 16 17 Obese patients are more at risk of various mental health conditions, such as anxiety and depression. Obesity is also the primary risk factor for the development of diabetes mellitus type 2. 18 In our cohort, diabetes was a significant predictor of lower PCS scores. This is also consistent with existing literature, individuals with diabetes have a higher vulnerability to frailty and diminished physical functioning, the cause of which is multifactorial. 19 Obesity and diabetes are chronic conditions, but adopting a healthy lifestyle can indirectly improve physical functioning and mental well-being over time. Educating patients afflicted with these conditions during consultation by outlining the expected risk and outcomes may be helpful. Enhancing weight and overall health is important for achieving improved functional outcomes in the long term. Obesity and diabetes were not shown to be predictive factors for diminished scores within the muscle flap cohort. Consequently, the consideration of opting for a muscle flap over a fasciocutaneous flap in these patients may be warranted. However, because to the limited number of participants in the muscle group, it is more difficult to distinguish differences between individuals, thereby precluding definitive recommendations.

Aside from the physical comorbidities obesity and diabetes, a diminished mental health was also found to be a negative predictor in patients treated with a fasciocutaneous flap. Patients with a mental score in the lower quartile of our cohort, scored relatively lower on the LEFS questionnaire. Wegener et al published data from the Lower Extremity Assessment Project Study: they conducted, however, a prospective study on solely patients with major lower extremity trauma but showed that the results were comparable. 20 The study involved a total of 327 participants and the findings indicated that increased levels of depression and anxiety after 6, 12, and 24 months were related with decreased scores of physical functioning. Furthermore, a correlation was observed between elevated pain degrees and diminished functionality after 6 and 12 months. Our cohort shows that these associations of mental health and pain with physical function persists up to a median follow-up of 4 years concurring with their results. All aspects of the biopsychosocial system should be considered when taking care of patients postoperatively to enhance their functioning and their quality of life.

Furthermore, in patients with a fasciocutaneous flap, a follow-up of 2 years or less was shown to have a negative predictive value for functioning measured by LEFS in comparison to a follow-up of more than 2 years. After this 2-year period, patients continue to improve in functionality. This could be explained by the fact that patients seem to cope and acquire improved use of their reconstructed extremity. This information might be included in patient consultation, highlighting that the postoperative recovery period is lengthy and comprehensive.

The following variables did not predict patient-reported outcome scores: gender, smoking status, hypertension, cardiovascular disease, primary or secondary surgery, surgery level, oncologic defect, traumatic defect, and recent or past trauma. Interestingly, individuals treated for a traumatic defect showed comparable functional outcomes to patients with a nontraumatic etiology.

The study was limited by the retrospective design and therefore unavoidable incomplete data retrieval. Total flap loss was not observed in either group. A total of 15 patients were included who underwent secondary reconstruction, after failure of previous flaps, or to address complications or deficiencies arising after the initial surgery. This study solely assessed data on the final reconstruction, thereby resulting in an underestimation of the number of complications, particularly total flap loss. The group patients undergoing lower extremity reconstruction and the etiology of the defect were quite heterogeneous. The study may be limited by the impact of surgeons' preference on reconstructive decision-making, despite comparable study populations among the participating centers. Distribution of patients among both groups was uneven: only 31 patients receiving muscle flaps compared with 69 fasciocutaneous flaps. Due to the limited numbers in the muscle group, it will be more difficult to find differences between individuals. This may explain why only pain was identified as a risk factor in the multivariate analysis. Lastly, we acknowledge the potential impact of the weight-bearing aspect of heel and foot reconstructions on postoperative scores. However, because to limited data, we were unable to explore this.

## Conclusion

After microsurgical soft tissue coverage of the lower extremity, patients treated with a free fasciocutaneous and muscle flaps experience similar levels of functionality and quality of life. Patients with pain generally score lower on functional and mental outcomes. These patients should therefore be referred to a pain management specialist at an early stage postoperatively. Other factors that were associated with decreased outcomes and therefore must be taken into account, include obesity, diabetes, lower mental health, and a follow-up of less than 2 years. Based on our findings, we advise patients to be treated in a holistic manner considering physical comorbidities, subjective pain, and mental condition. These factors will significantly impact their functionality and therefore quality of life.
